# Toxicity of Naphthenic Acids on the Chlorophyll Fluorescence Parameters and Antioxidant Enzyme Activity of *Heterosigma akashiwo*

**DOI:** 10.3390/antiox10101582

**Published:** 2021-10-08

**Authors:** Huanxin Zhang, Yumiao Zhou, Qiang Kong, Wenlong Dong, Zhihao Lin

**Affiliations:** 1 College of Geography and Environment, Shandong Normal University, Jinan 250000, China; zhouyumiao0802@163.com (Y.Z.); kongqiang0531@hotmail.com (Q.K.); 2 Shandong Marine Forecast and Hazard Mitigation Service, Qingdao 266104, China; dongwenlong529@163.com; 3 College of Marine Life Sciences, Ocean University of China, Qingdao 266100, China; linzhihao@stu.ouc.edu.cn

**Keywords:** naphthenic acids (NAs), *Heterosigma akashiwo* (*H. akashiwo*), hormesis, antioxidant system, photosynthesis, cell morphology

## Abstract

Petroleum hydrocarbons can serve as a carbon source for marine phytoplankton; so, marine high-acid crude oil pollution events are likely to result in algal outbreaks or harmful algal blooms (HABs) in surface waters. Naphthenic acids (NAs) are the primary acidic component of crude oil, and red tide is of great concern due to its high diffusivity and strong destructive properties. It is important to study the mechanism of the toxic effect of NAs on the typical red tide algae, *Heterosigma akashiwo*, for the balance and stability of marine algae. The mechanism of NAs’ damage effect was investigated in terms of the antioxidant enzyme activity, cell number, the chlorophyll positive fluorescence parameters, and the cell morphology of microalgae. Experiments confirmed the hormesis of low-concentration (0.5, 2, and 4 mg/L) NAs on *Heterosigma akashiwo*, and the indicators of high-concentration (8 and 16 mg/L) NA exposures showed inhibition. In this study, the toxic effect of NAs on the target organism showed a clear concentration–dose relationship. The 16 mg/L NAs stress caused severe damage to the morphology and structure of the target biological cells in a short time (96 h), and the population growth decreased. The target organisms showed a staged oxidative stress response to NAs. The behavior in the low-concentration treatment groups showed toxicant excitatory effects on the photosynthetic efficiency and antioxidant enzyme activity of the target organisms. This study provides theoretical and practical data for the development of an important toxicological model of the toxicant’s excitement effects and antioxidant defense mechanisms. In addition, it provides prospective research data for the prediction and avoidance of ecological risk from NA pollution in marine environments.

## 1. Introduction

The frequent occurrence of oil spills in coastal areas makes marine crude oil pollution an important environmental problem [1]. According to the current domestic demand, the use of high-acid crude oil is increasing. The current production of high-acid crude oil accounts for approximately 5% of the total global crude oil production each year and is increasing at an annual rate of 0.3% on average [2]. Naphthenic acids (NAs) are the primary acid component in high-acid crude oil, and its weight ratio in crude oil is 1–2% [3,4]. Petroleum spills have resulted in large amounts of NAs entering the environment, and they are now being found in environmental media such as groundwater, rivers, lakes, and sediments. In addition, the compounds have been detected in aquatic organisms, such as freshwater fish [5,6,7,8].

As a new pollutant, NAs have caused widespread concern due to their high level of ecotoxicity [9]. The formula of this compound is C_n_H_2n+z_O_2_, where n is the number of carbon atoms, and z is zero or a negatively even integer that refers to the loss of a hydrogen [10]. The ecotoxicity of NAs to terrestrial ecosystems, such as rivers and soil, has been confirmed [11,12], and there is evidence that NAs show stronger toxicity in aquatic environments [13]. Compared with environmental media, such as rivers and lakes, the hydrophilic carboxyl group makes NAs more soluble in slightly alkaline seawater, and it has been concluded that NAs will have a faster diffusion rate and wider spread in crude oil-contaminated seawater [14]. It has been confirmed that NAs are an important factor that influence the composition of marine phytoplankton communities [15]. However, there have been few studies that have investigated the response mechanism of their toxic effects. Existing reports have primarily focused on the impact of oil sand tailing sewage leakage on the growth of the phytoplankton population and the screening of phytoplankton with potential repair. One study showed that the spillage of oil sand tailing wastewater had no appreciable effect on the growth of phytoplankton blooms when the NA content was less than 6.5 mg/L. However, the population growth of phytoplankton was significantly suppressed as the NA levels increased [16]. Twenty-one phytoplankton species with potential remediation value for oil sand tailing wastewater were targeted by the screening. NAs have been shown to significantly inhibit the growth of 14 phytoplankton species [17]. 

Phytoplankton are the primary producers in marine ecosystems and the foundation of the structure and function of marine ecosystems [18]. The proliferation of an oil spill can cause harmful algal blooms (HABs) to break out, and HABs are more likely to occur in coastal and port areas due to natural and anthropogenic influences [19,20]. Previous studies have shown that, to a certain extent, petroleum hydrocarbon pollutants can cause some single-celled phytoplankton to explosively become dominant marine species, thereby triggering red tides [21,22]. *Heterosigma akashiwo* (*H. akashiwo*), the typical red tide microalgae, is the primary phytoplankton that triggers red tide [23]. The toxins secreted by red tide algae can directly cause the death of marine organisms, and they can even cause human poisoning by spreading through the food chain [21]. Red tide algae can absorb and utilize NAs as their main carbon source [15], and this has a significant impact on nutrient transport, productivity levels, and ocean carbon cycles within an ecosystem [24]. Currently, there has been very little research regarding the ecotoxicity of NAs to marine ecosystems, and this is necessary to strengthen the research on NA pollution in marine environments.

Our earlier studies demonstrated a time and concentration dependent toxic strain effect of NAs on marine microalgae, and microalgae exhibited growth and development toxicity due to the effect on their photosynthesis efficiency and cell morphologies [25]. However, there does exist a short-term (96 h) stress to *H. akashiwo* at different concentrations of NAs, but the resulting individual and community toxicity response has not yet been reported. Therefore, in the present study, we examine their effects on *H. akashiwo* at different times under different concentrations of NA stress. The indicators of stress include the population dynamic growth curve, cell morphology, photosynthesis parameters, and typical antioxidant enzyme activities, and these are utilized to clarify the mechanism of NA toxicity. This study comprehensively analyzes the toxic effects of NAs on *H. akashiwo* from four perspectives in an effort to provide important reference data to maintain the balance and stability of entire marine ecosystems.

## 2. Materials and Methods

### 2.1. Experimental Algae and Chemicals

The *H. akashiwo* strain, CCMA369, was obtained from the Marine Ecology Laboratory, the Ocean University of China. NAs (CAS No.1338-24-5, Catalogue: 70340, >97% pure) were obtained from Sigma-Aldrich (Cleveland, OH, USA). The other chemicals used in this study were of analytical grade and were obtained commercially.

### 2.2. Culture and Exposure of Microalgae

The microalgae were inoculated in 500 mL of BG11 medium under the following conditions: light intensity of 200 lux, a ratio of light to dark of 12:12 h, and a temperature of 19 ± 1 °C. The conical flasks were shaken three times a day. The same volumes of medium and algae were added during the early stationary phase to each 250 mL conical flask when the algae were in the exponential growth phase, making the concentration of algal cells approximately (19.166 ± 1.510) × 10^3^ mL^−1^. The NAs (the concentrations of the final solutions were 0, 0.5, 2, 4, 8, and 16 mg/L) were then exposed separately. For each group, the experiment was repeated three times. After shaking, the samples were incubated for 96 h in a light incubation room, and the indices were measured once every 24 h.

### 2.3. Dynamic Monitoring of the Microalgae Population

Six NA mass concentration gradients were established: 0, 0.5, 2, 4, 8, and 16 mg/L; and 0 mg/L was established as a blank control group. The experiments were repeated three times for each group. After the microalgae inoculation, the algae were sampled at 24, 48, 72, and 96 h. Algal growth measurements were performed. For each measurement of the algal density, algae were extracted using an algal solution. A total of 0.5 mL of the algal solution was used to fix the algal cells with Luger’s reagent, and then the cell density was calculated by measuring with a blood cell counting plate under a light microscope. The results are expressed as mean ± SD. The above operations were all conducted on an ultra-clean workbench [26].

### 2.4. Electron Microscopy Analysis

The cell morphology of *H. akashiwo* was studied using scanning electron microscopy (SEM) after exposure to 16 mg/L of NAs. Cells from the control and treatment groups were collected on day four, and a 2.5% solution of glutaraldehyde was added to the post-treatment samples, which were fixed at 4 °C overnight. Then, the fixative was emptied, and the specimens were treated with a phosphate buffer solution (PBS, 0.1 M, pH = 7.0) and washed three times for 15 min each. The samples were then held stationary in a 1% osmium solution for 1–2 h and rinsed with PBS for 15 min three times. The samples were dehydrated and treated with each concentration for 15 min. The samples were then treated two times with 100% ethanol for up to 20 min each. Then, the samples were dried in a critical point dryer (HCP-2, Hitachi Electronic Instruments, Tokyo, Japan), and the coating process was performed in an ion sputterer (E-1010, Hitachi, Japan). Scanning was then conducted at 10 kV using a Hitachi S4800 FE-SEM with a field emitted SEM (Hitachi, Japan) [27].

### 2.5. Determination of the Chlorophyll Fluorescence Parameters

For the measurement of the chlorophyll fluorescence parameters, Water-PAM (Walz, Effeltrieh, Germany) was conducted, and the results represent the photosynthetic performance of *H. akashiwo*. Fv/Fm (PSII primary light energy conversion efficiency), ETR (apparent electron transport rate, potential activity), yield (the effective PSII quantum yield), and qP (coefficient of photochemical quenching) were used to characterize chlorophyll fluorescence parameter changes. The samples were subjected to the dark for 15 min prior to exposure to LED light pulses for determination of the parameter [28].

### 2.6. Determination of the SOD (Superoxide Dismutase) and CAT (Catalase) Activities

At 0, 24, 48, 72, and 96 h of the experiment, 40 mL of the algal solution was taken from each group of algal cultures and centrifuged at 4500× *g* for 15 min. The algal cells were then collected and resuspended by adding 0.01 mol/L of PBS and were sonicated at 4 °C for 5 min using a cell crusher. After complete cell disruption, the samples were centrifuged at 5000× *g* at 4 °C for 15 min, and the supernatant was used to determine the SOD and CAT of the samples using a microplate reader (Multiskan FC, Thermo Fisher, Waltham, MA, USA). The assay was performed using SOD (19160, Sigma-Aldrich, St. Louis, MO, USA) activity and CAT (11363727001, Sigma-Aldrich, St. Louis, MO, USA) activity assay kits [29,30]. Three parallel experiments were performed for each group.

### 2.7. Data Analysis

The experimental data were analyzed using IBM SPSS 22.0 statistical software (SPSS, Inc., Chicago, IL, USA). A one-way ANOVA was utilized with *p* < 0.05 defined as a significant difference. A Levene’s test was first used to test the homogeneity of the data, and the Shapiro–Wilk test was used to test the normality of the data. Dunnett’s T3 was used to compare the mean between groups when there was a variation between groups and the variance was different. Multiple comparison tests were then performed (Student–Newman–Keuls post hoc multiple comparison test and Duncan’s post hoc test) [31]. The data were plotted in GraphPad Prism 5.0 software (GraphPad Inc., Chicago, IL, USA).

## 3. Results

### 3.1. Effects on the Growth of H. akashiwo

**Figure 1:**
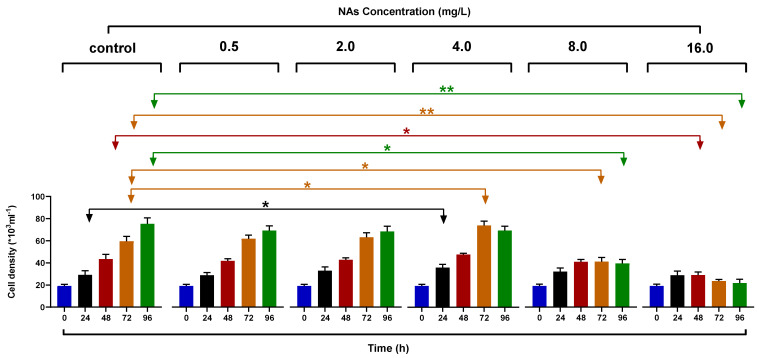
The growth curve of *Heterosigma akashiwo* exposed to NAs at different concentrations with different times. Data are expressed as mean ± SD. * and ** indicate statistical differences between the different NAs treatment groups at the same time, assessed according to one-way ANOVA and Dunnett’s T3 post hoc test (*p* < 0.05). * indicates *p* < 0.05. ** indicates *p* < 0.01.

For the control group, all of the microalgae were in the exponential growth phase, and their corresponding initial density was (19.166 ± 1.510) × 10^3^ mL^−1^. The cell density of the experimental group at 4 mg/L of NAs reached (73.853 ± 3.841) × 10^3^ mL^−1^, which was significantly higher than that of the control group ((59.510 ± 4.470) × 10^3^ mL^−1^) in 72 h after exposure, and the difference was significant (*p* < 0.05). The cell density of the 16 mg/L NAs stress reached (23.557 ± 1.565) × 10^3^ mL^−1^, which was extremely significantly lower than that of the control group (*p* < 0.01), as shown in Figure 1. The mean cell density increased to (75.333 ± 5.346) × 10^3^ mL^−1^ in the control group during the experimental period, and all of the NA concentrations were compared, as shown in Figure S1. Additionally, the microalgae cell density changed. There was a significant difference between the microalgae in the treatment group and the stress group after 48 h (*p* < 0.05). Overall, the degree of inhibition continued to increase with an increase in the concentration of NAs and time. In summary, the level of inhibition continued to increase with NA concentration and time. This indicated that the stress effect of NAs on the microalgae was heightened by an increase in the test time and an increased concentration of the stressor.

### 3.2. Effects on Cellular Morphology

**Figure 2:**
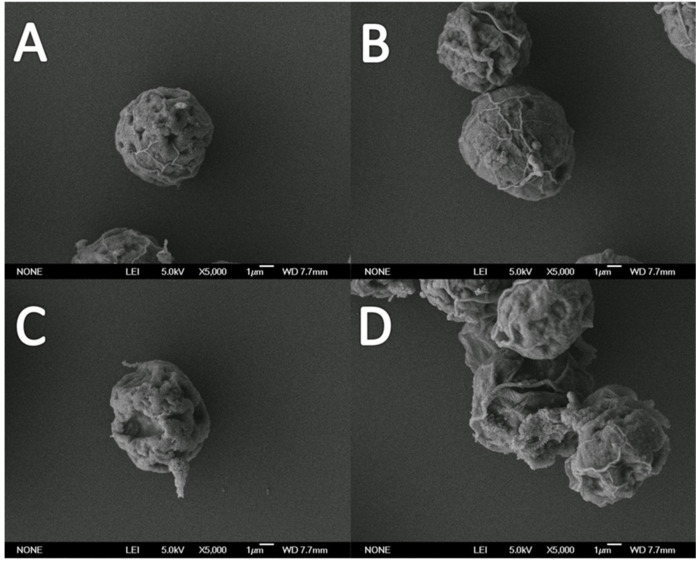
The SEM morphology of *H. akashiwo* exposed to NAs for 96 h. (**A**) The control at 0 h; (**B**) the control at 96 h; (**C**) *H. akashiwo* monomer; and (**D**) *H. akashiwo* aggregate exposed to 16 mg/L of NAs for 96 h.

In the control group, the cells were intact at 0 h and were round and full. Because they were in the exponential growth phase, the number of cells increased significantly in the visual field, as shown in Figure 2A. For the 16 mg/L NAs treatment group, the cell morphology showed a significant change compared with the control group. The individual cells were severely depressed, the cell body was severely damaged, and the cytoplasm was shed (Figure 2C). The control group had normal cell morphology at 96 h, but the cell surface was poor in smoothness. However, the whole cell had a good fullness. When the microalgae grew to the late logarithmic growth phase, there was no significant difference in cell morphology compared to the inoculation stage, but the total amount of cells was greatly increased in the visual field (Figure 2B). For the 16 mg/L NAs treatment group, the cell morphology showed significant changes with respect to the control group. In addition, the cells produced various deformations, the fullness was greatly reduced, and some cell structures were damaged and ruptured. When the concentration of NAs was equal to 16 mg/L, the NA-induced stress still had a serious negative impact on the surface morphology of the cells (Figure 2D).

### 3.3. Effects on the ChlF (Chlorophyll Fluorescence) Parameters

**Figure 3:**
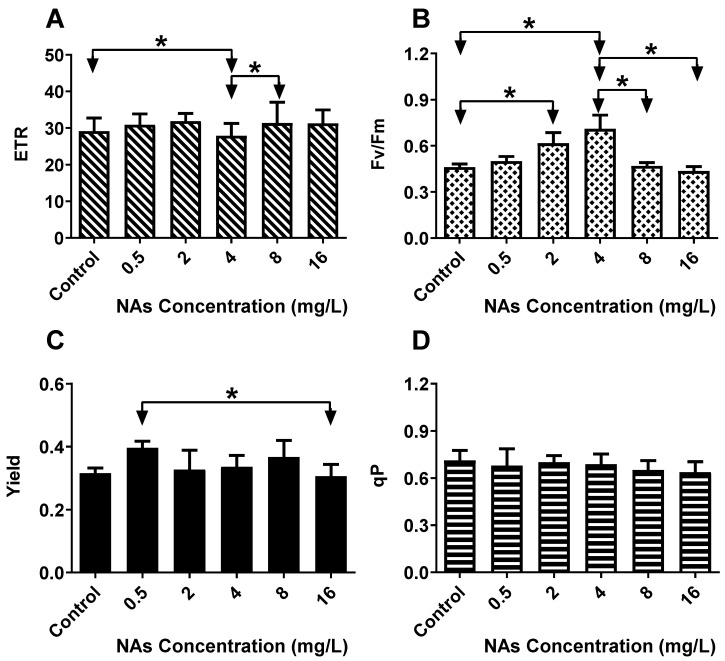
The changes in the ChlF parameters: (**A**) ETR, (**B**) Fv/Fm, (**C**) yield, and (**D**) qP of *H. akashiwo* exposed to NAs at different concentrations for 96 h. Data are expressed as mean ± SD. * indicates statistical differences between the different NAs treatment groups, evaluated according to one-way ANOVA and Dunnett’s T3 post hoc test (*p* < 0.05). * indicates *p* < 0.05.

As shown in Figure 3A for the ETR, when the NAs’ concentration was in the range of 0–2 mg/L and 4–16 mg/L at 96 h, the ETR of each group was compared, and there was an increasing trend. The most significant decrease in ETR was observed in the 4 mg/L NAs treatment group (27.90 ± 3.36), which was 95.77% of that of the control group (29.13 ± 3.61). The ETR value of the 8 mg/L NAs treated group was 31.37 ± 5.70, which was 107.70% of that of the 4 mg/L NAs treatment group. Fv/Fm decreased significantly under stress conditions, turning it into a useful probe-and-index. As shown in Figure 3B for the Fv/Fm, at 96 h, when the NA concentration was in the range of 2–4 mg/L, the Fv/Fm of the two groups were compared, and both the groups showed increasing trends. In addition, this difference was significant (*p* < 0.05). When the concentration of NAs was 8–16 mg/L, the measured value of Fv/Fm in the treatment group was reduced. With an increase in the NA concentration, the Fv/Fm first promoted and then inhibited growth. Fv/Fm, the photochemical efficiency, was proportional to the quantum yield of PSII photochemistry and was highly related to the quantum yield of net photosynthesis. As shown in Figure 3C for the yield, at 96 h, when the NA concentration was in the range of 0.5–8 mg/L, the comparison between the two groups primarily showed an increasing trend. The NAs’ toxicity-promoting effect in the 0.5 mg/L group was significantly higher than in the other groups, with yield values reaching 0.40 ± 0.02. When the NA concentration was equal to 8 and 16 mg/L, the treatment group showed a continuous decrease, and the degree of yield inhibition was more significant in the 16 mg/L treatment group. In addition, there was no significant difference in the other groups at this time (*p* > 0.05). As shown in Figure 3D for the qP, at 96 h, when the NA concentration was in the range of 0.5–16 mg/L, the qP of the exposed groups and the control group were compared. The results show that the qP revealed an obvious concentration–effect relationship with increasing NA concentration, and the qP maximum value in the NAs exposed group was 0.70 ± 0.04, which was 98.4% of that of the control group (0.711 ± 0.06). The greatest decrease in qP was observed in the 16 mg/L group (0.64 ± 0.07), which was 89.59% of that of the control group.

### 3.4. Effects on the SOD and CAT

**Figure 4:**
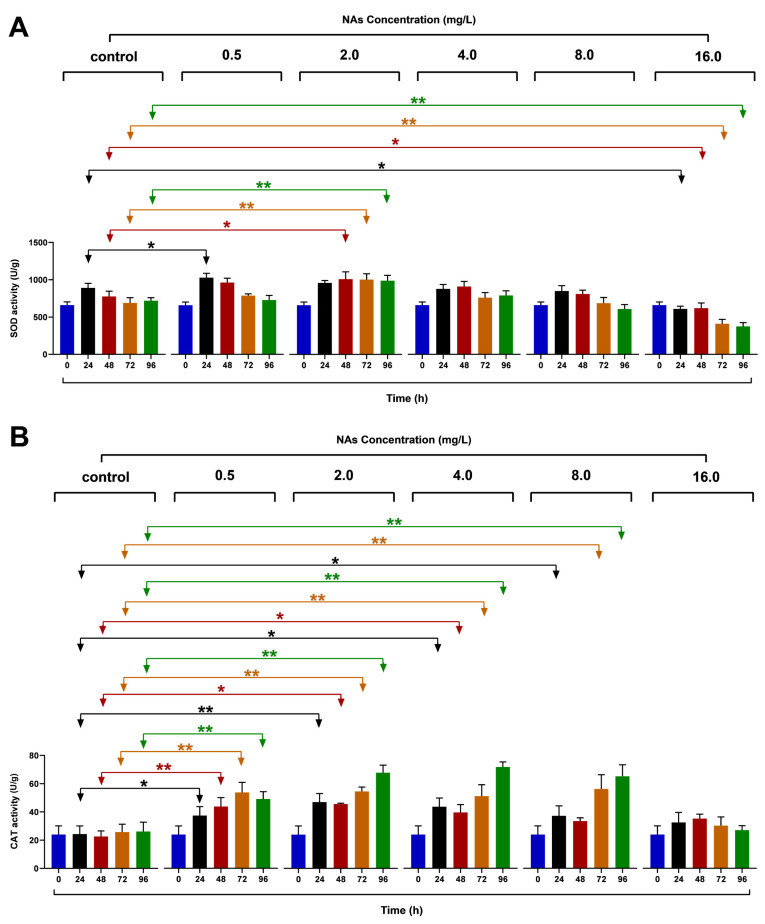
(**A**) The changes in the SOD of *H. akashiwo* exposed to NAs at different concentrations for 96 h. (**B**) The changes in the CAT of *H. akashiwo* exposed to NAs at different concentrations for 96 h. Data are expressed as mean ± SD. * and ** indicate statistical differences between the different NAs treatment groups at the same time, assessed according to one-way ANOVA and Dunnett’s T3 post hoc test (*p* < 0.05). * indicates *p* < 0.05. ** indicates *p* < 0.01.

The low-concentration (0.5 mg/L) treated groups showed an increasing trend in the SOD and CAT activity at the early stage of 24 h NAs exposure with remarkable differences (*p* < 0.05), as shown in Figure 4. The coefficients of variation for the results of the high-concentration (16 mg/L) NAs treatment groups were 11.13%, 14.39%, and 13.30% (48, 72, and 96 h), and the degree of change was more significant compared to the control group (9.15%, 10.00%, and 5.42%) at the same time. With an increase in time, in the 72 and 96 h phase, the SOD activity of the low-concentration NAs treatment group (2 mg/L) was 160.28% and 137.50% of that of the control group, and there was an extremely significant difference (*p* < 0.01). However, the high-concentration treatment group showed a downward trend in the SOD and CAT activity and declined at different rates (Figure 4). In addition, the SOD activities of the experimental group treated with 16 mg/L NAs were 52.22% of the control group, and the difference was highly significant (*p* < 0.01). However, the CAT activity was 103.71% of the control group under the same treatment conditions, and the change effect was not as obvious as that in the SOD activity.

As shown in Figure 4A, the SOD activity increased in the 0.5 and 2 mg/L NAs treatment groups compared to the control group during the experimental period and began to decrease with increasing NA concentrations. The effect of the variation was more pronounced in the low-concentration (0.5–4 mg/L), short duration treatment group. For example, the 2 mg/L NAs treatment group showed a significant difference in SOD activity at 48 h (*p* < 0.05), while the 72 and 96 h time points showed highly significant differences (*p* < 0.01). A decreasing trend in the SOD activity was observed when the NA concentration was greater than 4 mg/L, and this change was similar to the CAT activity. The distinction from changes in the CAT activity was that the change in the SOD activity in the 16 mg/L NAs treatment group was remarkably lower than in the other treatment groups (*p* < 0.01). Moreover, the SOD activity decreased gradually with increasing time (48, 72, and 96 h) in the same NAs treatment groups (2.0, 4.0, and 8.0 mg/L), but the CAT activity showed an increasing trend. As shown in Figure 4B, the CAT activity of *H. akashiwo* in the 0.5–8 mg/L treatment groups showed an increasing trend, and the CAT activity in the 16 mg/L treatment group decreased slightly. In particular, 24 and 96 h after NAs exposure, the CAT activity showed a significant promotion at low concentration (0.5–8 mg/L), while at high concentration (16 mg/L) it was relatively inhibited.

## 4. Discussion

According to the research data of this experiment, a low NA concentration in seawater culture promoted *H. akashiwo* population growth in a short time. Low concentrations of organic pollutants promote the growth and development of organisms in a short period of time. This toxic effect is called hormesis. Hormesis is a physiological response of an organism to a toxic substance with an adaptive mechanism. Within the range of different toxicant doses and toxicities, the organism itself has different toxic dose responses [32]. This reaction is primarily determined by the organism’s own toxicological adaptation mechanism and the various tolerance stages of the physiological and biochemical processes. It is generally manifested in the impairment of physiological and biochemical processes and growth of the organism itself at high doses, and developmental reproduction is inhibited. However, when the dose of a poison is at a certain low concentration, there is a stimulating effect on the physiological and biochemical processes, which has a positive effect on its growth and development [33].

According to previous studies, low concentration doses of pollutants promote the growth of phytoplankton such as microalgae [34]. According to this study, the low concentration of α-naphthol contained in the material was the carbon source required for the growth of chlorella, which was released in seawater, obtained by the algal species and utilized for its normal growth and development [35]. During the process, some plankton in the ocean were selected by the natural environment during the long-term evolution of the species. In order to improve the reproductive ability and adaptation mechanism of the plankton under different natural stress conditions, a special adaptation mechanism was formed. In the case of damage and growth and the development of normal physiological and biochemical processes in the environment, control and formation of new adaptation mechanisms are achieved in a short period of time [36].

The results of this study showed that NAs have a significant effect on the photosynthesis of *H. akashiwo*, and its effect on photosynthesis was closely related to its concentration. The effects of NAs on photosynthesis in plants at different concentrations are quite different. At low concentrations it promotes photosynthesis in plants, and at higher concentrations it can play a destabilizing role. Therefore, we can conclude that the stimulating effect of the dose of NAs on the plant body was due to the fact that a small amount of NAs increased the level of photosynthesis of the plant and promoted the continuous conversion of other energy sources, thereby enhancing the metabolic function of the plant body [33]. This caused an indirect effect that allowed the microalgae to grow quickly. Low concentrations of NAs can increase photosynthesis, increase chlorophyll content, enhance protein synthesis efficiency, and regulate various physiological functions, thereby stimulating population growth. A low concentration of NAs increases Fv/Fm. Stimulated by low concentrations of NAs, the photosynthesis of the microalgae was enhanced. This promoted the maximum light energy conversion efficiency and actual light energy capture efficiency, improved the photosynthetic electron transport efficiency, and increased the number of electrons in photosynthesis.

For the petroleum-based pollutant NAs, two antioxidant enzymes contained in the microalgae can also exhibit stress activity, thereby reducing various hazards caused by stress. This is because NAs promote redox reactions to a large extent, following which abundant active oxygen is formed. The antioxidation defense system of microalgae can effectively remove a large amount of free radicals, thereby avoiding damage to the body. In this study and a related study, the effects of α-naphthol stress on the activity of chlorella and the effect of petroleum hydrocarbons on *Nannochloropsis* and 1-hexyl-3-methylimidazolium on the antioxidant enzyme activities of *Scenedesmus obliquus* were analyzed [37]. The results were highly consistent. However, there were differences in the extent of enzyme activity increase in the various microalgae. Not only is SOD a superoxide anion scavenger enzyme, but it is also a major hydrogen peroxide-producing enzyme with an important role in biological antioxidant systems [38]. Some microalgae had reduced enzyme activity when the NA dose was relatively low, and some species still exhibited ideal SOD after prolonged exposure. The CAT activity showed excellent tolerance, and the SOD of red tide algae had a very good tolerance to NAs. The most significant difference between CAT and other enzymes is that they do not require reducing agents for the disproportionation reactions they catalyze [39]. For CAT, *H. akashiwo* showed some tolerance to NAs, but as the test time increased the tolerance gradually decreased. Its CAT was in the lower concentration range, with activity enhancement as the core. In addition, no decrease in activity was observed. This indicates both enhancement of CAT activity and reduction of pollution under the adverse condition of NA toxicity. Under NA stress, *H. akashiwo* increased active oxygen in order to avoid an attack of biological macromolecules in the body. Research has shown that the CAT of *Heterosigma erythraea* (*H. erythraea*) also showed stress activity during the early stage of NAs exposure. However, with a continuous increase in exposure time, the CAT activity was significantly reduced and inhibited; hence, it was tolerated [40,41].

NAs at low concentrations were previously reported to promote the growth of *H. akashiwo*, while NAs at high concentrations inhibited the growth of *H. akashiwo*. It was confirmed that low concentrations of crude oil pollutants have a stimulating effect on marine microalgae during a short time period and they have a significant inhibitory effect on various marine microalgae at high concentrations (30–60 mg/L) [30,42,43]. According to Almeda R et al., crude oil (1, 5, and 25 μL/L) can stimulate the population growth of *Prorocentrum texanum* [21]. A study of the toxic effects of petroleum hydrocarbons was not only conducted in a laboratory but also in an ecological environment [44]. In the Nile River Basin, Elsheekh used enclosure experiments to study the effects of petroleum pollutants on dominant species of algae (cyanobacteria and diatoms), further confirming that various petroleum pollutants can stimulate the growth of phytoplankton [45]. Petroleum hydrocarbons have been found to exhibit low-promotion and high-suppressing effects on marine phytoplankton. This is roughly inferred from the complexity of known components of contaminants contained in petroleum and their inclusions. This is because hydrocarbons in NAs and nitrogen-containing compounds, as sources of organic carbon and nitrogen necessary for the growth of phytoplankton, can be used as nutrients, and organisms show a considerable degree of compensation mechanism action when subjected to toxic stress [46,47]. The toxic substances contained in NAs will cause some damage to the physiological and biochemical processes of phytoplankton cells with the increase in dose poisoning and the prolongation of the poisoning time; ultimately, this will cause substantial damage to phytoplankton cells over a certain period of time. This short-term, low-concentration promotion and high-concentration inhibition of NAs provide more entry points for studying long-term oil pollution hazards to ecosystems. High concentrations of organic pollutants can inhibit the physiological mechanisms of the organisms to different degrees, causing the cell membrane to break. Then, the harmful substances in the tested pollutants are fully inputted into the organism, causing serious toxicity damage. According to previous studies, a high concentration of benzopyrene, a petroleum pollutant, severely damages the photosynthesis process of chlorella, causing severe damage to the photosynthesis organs of algae and serious obstruction of the photosynthetic electron transport and photosynthetic nitrogen fixation processes of algae [48]. This can cause serious damage to their growth and development. The toxic effects of NAs on marine microalgae will cause some microalgae to multiply and become dominant species, thus further changing the population structure. Low concentrations of NAs have a stimulating effect on the growth and physiological characteristics of marine microalgae. Some may be stimulated to grow and become the dominant species. Therefore, the rapid propagation of harmful marine microalgae, such as red tide algae, can lead to changes in the structure of the ocean population, leading to the formation of red tides that ultimately lead to the destruction of marine ecosystems.

## 5. Conclusions

We investigated the effect of five NA exposure levels (0.5, 2, 4, 8, and 16 mg/L) on the algae *H. akashiwo*. NAs at low concentrations (0.5, 2, and 4 mg/L) promoted the physiological and biochemical indices (the population growth, SOD and CAT activity, and ChlF parameters) of *H. akashiwo* over a short period of time (48 h). However, the total number of cells increased significantly with the mildly damaged cell structure of *H. akashiwo*. This disruptive growth was attributed to the stress effect of *H. akashiwo* cells adapting to a mutant environment. The cell structures were completely damaged and broken after NA exposure at high concentration (8 and 16 mg/L). The toxic effects of NAs on *H. akashiwo* showed a clear concentration–effect relationship. This study elucidated the toxic effects on *H. akashiwo* at different time points under different concentrations of NA stress from multiple perspectives. This study provides prospective research data for ecological risk predictions and avoidance of red tide due to NAs in marine environments.

## Data Availability

Data are contained within the article and Supplementary Material.

## Supplementary Materials

The Supplementary Materials can be found on https://www.mdpi.com/article/10.3390/antiox10101582/s1. Figure S1. Relative growth rates of *Heterosigma akashiwo* under different concentrations (0.5, 2, 4, 8 and 16 mg/L) of NAs exposure on different times (24, 48, 72 and 96 h).
