# Supplementary material for: Toxicity of Naphthenic Acids on the Chlorophyll Fluorescence Parameters and Antioxidant Enzyme Activity of Heterosigma akashiwo
Source: Antioxidants (Basel). 2021 Oct 8;10(10):1582. doi: 10.3390/antiox10101582 (PMC8533473; doi:10.3390/antiox10101582)
Supplement: Supplementary file 1 [file antioxidants-10-01582-s001.zip › antioxidants-1378296-supplementary.pdf]

SUPPORTING INFORMATION

Toxicity of Naphthenic Acids on the Chlorophyll Fluorescence Parameters and Antioxidant Enzyme Activity of *Heterosigma akashiwo*

Huanxin Zhang [1, †, *], Yumiao Zhou [1, †], Qiang Kong [1], Wenlong Dong [2] and Zhihao Lin [3]

[1] College of Geography and Environment, Shandong Normal University, Jinan, 250000, China; qshdzhhx@126.com (H.Z.); zhouyumiao0802@163.com (Y.Z.); kongqiang0531@hotmail.com (Q.K.)

[2] Shandong Marine Forecast and Hazard Mitigation Service, Qingdao 266104, China; dongwenlong529@163.com (W.D.)

[3] College of Marine Life Sciences, Ocean University of China, Qingdao 266100, China; linzhihao@stu.ouc.edu.cn (Z.L.)

* Correspondence: qshdzhhx@126.com; Tel: +86-157-6225-6586.

[†] These authors contributed equally to this work.

2 pages, 1 figures

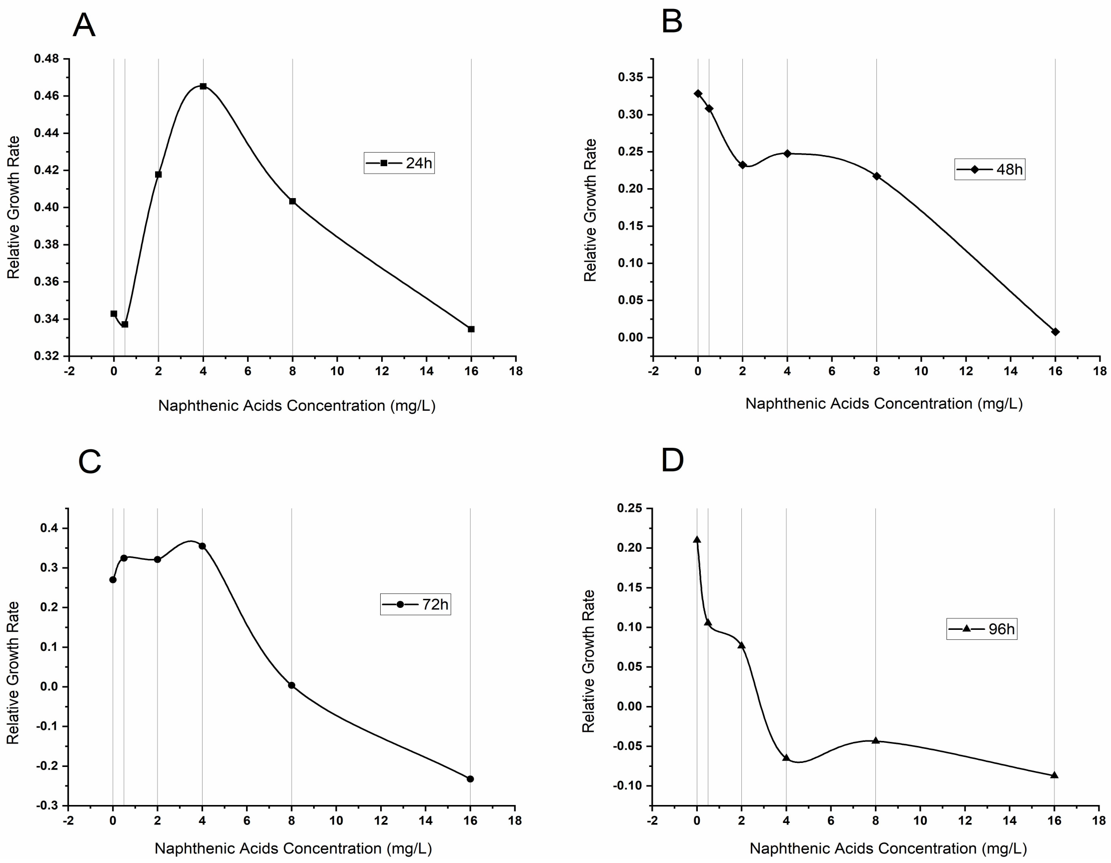

Figure. S1. Relative growth rates of *Heterosigma akashiwo* under different concentrations (0.5, 2, 4, 8 and 16 mg/L) of NAs exposure on different times (24, 48, 72 and 96 h).
